# Estimating variability in downwelling surface shortwave radiation in a tropical highland environment

**DOI:** 10.1371/journal.pone.0211220

**Published:** 2019-02-25

**Authors:** Stephanie Stettz, Benjamin F. Zaitchik, Dereje Ademe, Sintayehu Musie, Belay Simane

**Affiliations:** 1 Department of Earth & Planetary Sciences, Johns Hopkins University, Baltimore, Maryland, United States of America; 2 Debre Markos University, Debre Markos, Amhara, Ethiopia; 3 College of Development Studies, Addis Ababa University, Addis Ababa, Ethiopia; Universidade de Vigo, SPAIN

## Abstract

Surface incoming shortwave (solar) radiation data are an important component of many scientific analyses, but direct measurements are not commonly available. Estimates can be obtained from gridded meteorological analysis or reanalysis systems, such as the Global Data Assimilation Systems (GDAS) and Modern Era Retrospective Reanalysis System (MERRA-2), or calculated using empirical models dependent on meteorological variables such as air temperature. The purpose of this analysis was to compare multiple methods for estimating daily shortwave radiation in a tropical highland environment in Ethiopia. Direct solar radiation outputs of GDAS and MERRA-2, topographically corrected outputs of the two analysis systems, and empirically estimated solar radiation values calculated with the systems’ air temperature data were compared to see which produced the most reliable radiation values. GDAS appeared to underestimate the seasonal variability, resulting in low correlation (R^2^) with *in situ* data and large mean bias error (MBE). In comparison, MERRA-2 did not underestimate variability, but produced larger bias than the empirical model estimates. There was an improvement in correlation and reduction in MBE when using the GDAS air temperature predictions in the empirical model, but the opposite was true for MERRA-2. The empirical model using station air temperature data (*stationT*) produced the highest correlation across all four stations, with best performance at the lower elevation sites. The direct shortwave radiation outputs of MERRA-2 produced comparable correlation values, with larger R^2^ at stations at higher elevation. Topography possibly influenced these results, as MERRA-2 performed comparably to *stationT* at the stations in moderate terrain, but not in steeper terrain. This work can serve as a starting point for analyses in other tropical highland regions, where continuous in situ solar radiation data are rarely available.

## 1.0 Introduction

### 1.1 History & importance of solar radiation data

Data on incoming solar radiation at the surface are required for many applications, including surface energy balance analyses, generation of evaporation and transpiration estimates, and site selection for solar energy production. However, direct measurements of incoming solar radiation at the surface are rarely available, so estimates are frequently employed [1]. Many empirical models have been developed to produce shortwave radiation estimates. In some environments, relatively simple day of year models provide adequate performance [2, 3], using only astronomical and static geographical (latitude, longitude, topography) inputs. For other environments and applications, however, it is necessary to integrate some kind of meteorological predictors to the model, in the form of sunshine-hour or cloud cover data or as direct meteorological inputs of temperature, precipitation, humidity, and/or other relevant atmospheric conditions [4, 5].

The number of sunshine hours and cloud cover are intuitive inputs for a surface downwelling shortwave radiation estimate, but they are difficult to measure and are not commonly recorded at most weather stations [4]. Since daily minimum and maximum air temperature are almost always recorded by professional grade weather stations, many empirical models have been developed to estimate solar radiation as a function of air temperature [6, 7]. Temperature-based models work on the assumption that maximum temperature will decrease with reduced atmospheric transmissivity, and minimum air temperature will increase due to increased cloud cover [7]. Clear skies, in contrast, will result in higher maximum temperatures due to higher shortwave radiation, and minimum air temperatures will decrease due to lower atmospheric emissivity. The performance of different temperature-based estimates can vary quite widely in different environments. Even in cases where the estimates provided by different methods are relatively similar, even moderate differences in performance can have a significant impact when the radiation estimates are used as an input to a crop model [8].

Radiation estimates can also be obtained from surface meteorology analysis systems such as Global Data Assimilation System (GDAS) or Modern-Era Retrospective Analysis for Research and Applications (MERRA-2) [9, 10]. These systems assimilate a diverse suite of surface and remotely-sensed observations to advanced meteorological models, yielding gridded estimates of surface meteorology, including incoming solar radiation, that are continuous in space and time. The systems vary in the details of the atmospheric model used, the choice of assimilated observations and the assimilation algorithm, and the number of processes accounted for in the simulation system. In all reanalysis systems, however, surface incoming solar radiation is calculated as an outcome of atmospheric radiative transfer processes rather than estimated as a function of other near-surface variables. Previous work has shown that reanalyses products contain considerable uncertainty in the estimate of solar radiation at daily timescales [11], though no analysis has been performed in tropical highland regions.

A third class of radiation estimates are those derived from satellite observations (e.g., [12]). These methods make use of satellite observed upwelling shortwave radiation at the satellite, which, in combination with independent information on surface albedo, cloud properties, and atmospheric radiative transfer, can be used to estimate downwelling radiation at the surface. These methods are increasingly applied for solar radiation monitoring and have been leveraged for short-term radiation forecasts. These methods are not included in this study, as our focus is on comparing advanced reanalyses to station-based empirical methods in a highly cloud affected region, with an eye to applications in seasonal forecasts and climate projections.

### 1.2 Region of study: Northern Ethiopian Highlands

The Ethiopian Highlands are a densely populated, mostly rural region, dominated by smallholder subsistence agriculture. Local climate variability is extreme: steep, dissected topography and a dominance of convective precipitation processes mean that temperature, rainfall, and other meteorological conditions can vary significantly over distances of just a few kilometers. Meteorological monitoring under such conditions is a considerable challenge, and the importance of this challenge is underscored by the climate vulnerability of the local farming population [13, 14].

In this context, the importance of rainfall monitoring [15–17] and, to some extent, temperature monitoring [18, 19], have been considered at some length. Here, we consider the problem of estimating local incoming shortwave (solar) radiation. This variable is relevant for multiple applications in climate, agriculture, and energy analysis, and it stands out as a particularly important variable in that it is a required input to many process-based crop models. Publicly available *in situ* measurements of incoming shortwave radiation are almost completely absent in the Ethiopian Highlands, and steep topography makes it difficult to extrapolate from any single measurement site with confidence.

The objective of this study was thus to determine the best way to estimate daily-received solar radiation when there are no *in situ* measurements available. The direct output of meteorological analysis systems, topographically corrected meteorological analysis outputs, and empirically estimated solar radiation values calculated with meteorological analysis temperatures were compared to see which produced the most reliable results.

## 2.0 Materials & methods

### 2.1 Stations

**Fig 1:**
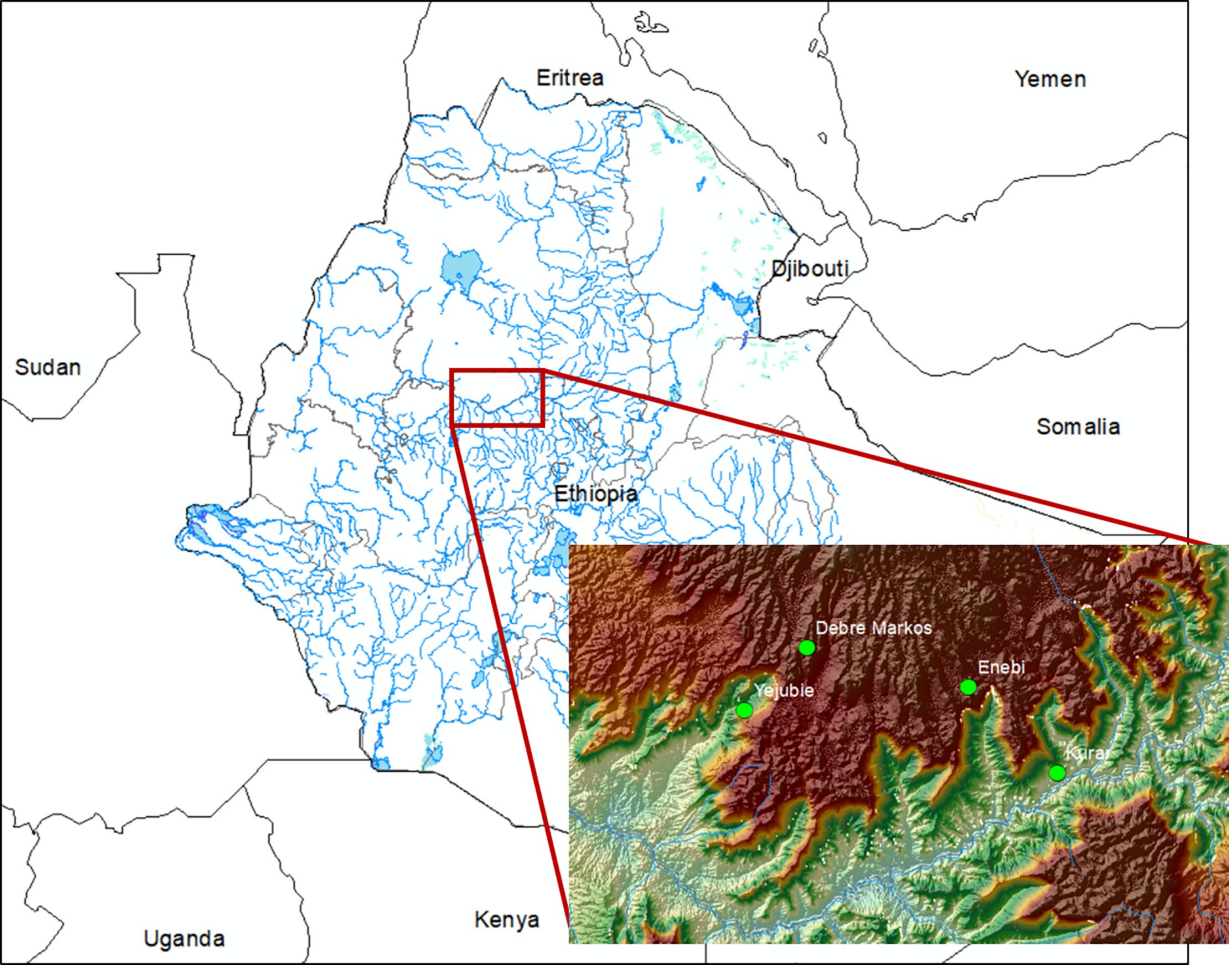
Map of Ethiopia and locations of sites within the Ethiopian Highlands.

Weather station air temperature and solar radiation data from four sites in the Ethiopian Highlands were used in this study. The Kurar station is located in the steepest terrain and the lowest elevation of the four sites, with the hottest recorded temperatures. The Debre Markos and Yejubie stations are located in the rolling hills, while the Enebi station is located on a flat plain (Fig 1). All data were collected using Davis Instruments Vantage Pro2 weather stations installed at 2 m height on tripods sited in the middle of open fields. A fifth station installed near the top of the mountain failed during data collection and is not included in this study.

Table 1 contains the latitude, longitude, elevation, and time periods of the station data available for each site. Inset shows topography, derived from NASA Shuttle Radar Topography Mission (SRTM) data and mapped using ESRI ArcMap 10.4.

**Table 1:** Latitude, longitude, elevation, and time periods of available data from each weather station.

Site name	Latitude	Longitude	Elevation (m)	Time period of recorded data
Kurar	10°06’00”	38°12’00”	1826	5/2/2014–11/3/2014, 1/1/2015–1/31/2015
Yejubie	10°12’00”	37°42’00”	2342	5/2/2014–10/15/2014, 1/27/2015–4/8/2015, 5/16/2015-6/20/2015
Enebi	10°14’17”	38°03’30”	2430	5/2/2014–7/14/2014, 8/5/2014–9/14/2014, 11/3/2014–1/28/2015, 4/2/2015–6/29/2015, 7/17/2015–8/13/2015, 1/14/2016–4/29/2016
Debre Markos	10°18’00”	37°48’00”	2448	6/6/2014–12/8/2014, 1/1/2015–5/29/2015, 8/29/2015–12/14/2015, 3/6/2016–6/21/2016

### 2.2. Model selection

Almorox [1] tested fifteen different solar radiation empirical models, categorized based on the primary meteorological variable used. The models were tested using weather station data from Spain, and the coefficient of determination (R^2^), root mean square error (RMSE), and mean bias error (MBE) was compared to evaluate which type of model made the most accurate solar radiation estimates. Almorox has also tested air temperature empirical models in other locations in Spain [20], Venezuela [21], and Argentina [4]. In this analysis in the Ethiopian Highlands, four of the temperature models discussed in the literature study were tested: Hargreaves & Samani [22], Bristow & Campbell [6], Donatelli & Campbell [23], and the Goodin [7] model. While it has been acknowledged that temperature-based models produce less accurate estimates than cloud or sunshine-based models [1], air temperature is more commonly measured at weather stations. The results showed that out of all the temperature-based models tested with the data from Spain, the Bristow & Campbell model produced the most accurate estimates [1]. This influenced the decision to test the Bristow & Campbell model [6] and its other forms (Donatelli & Campbell [23] and Goodin [7] models) with the Ethiopia air temperature data. The Hargreaves & Samani model [22] was also tested due to its simplicity. All of the methods tested in this study are process-based structured equations—i.e., the form of the relationship is fixed based on hypothesized and/or observed relationships between the meteorological predictors and the response variable. We note that in recent years a number of investigators have applied machine learning and data mining approaches to the problem of estimating shortwave radiation, and these new methods have shown good performance in several cases [24–27]. We choose to focus on the structured equations since they are established and relatively easily applied methods, and they provide a useful point of comparison to the globally available reanalysis products that we also evaluate.

### 2.3 Empirical models

We tested these models by using the *in situ* radiation and air temperature data to fit the parameters of each model in MATLAB R2016b on Mac OS X (using the *fminsearch* function), and generated shortwave radiation estimates with the four different temperature-based models. The maximum, minimum, mean, standard deviation, as well as the correlation (R^2^), root mean square error (RMSE), and mean bias error (MBE) were calculated to compare the performance of each model.

#### 2.3.1 Hargreaves & Samani model

The model is solely dependent on the difference between maximum and minimum daily air temperature [22], and can generate shortwave solar radiation (R_S_) estimates using the following equation:

$$R_{s}=R_{a}[A\sqrt{\left(T_{max}-T_{min}\right)}]$$ (1)

The extraterrestrial radiation (R_a_) is the intensity of radiation at the top of the atmosphere, and is estimated as a function of five variables: latitude (φ, radians), solar declination (δ), sunset hour angle (ω_s_), inverse-relative distance from Earth to the Sun (d_r_), Julian day (J) and the solar constant (G_sc_, 0.0820 MJ m^-2^ min^-1^). The extraterrestrial radiation, solar declination, sunset hour angle, and inverse-relative distance were calculated using Eqs 2 through 5 [28].

$$R_{a}=\frac{24(60)}{\pi }G_{sc}d_{r}[\omega_{s}\sin\left(\varphi \right)\sin\left(\delta \right)+\cos\left(\varphi \right)\cos\left(\delta \right)\sin\left(\omega_{s}\right)]$$ (2)

$$d_{r}=1+0.033cos(\frac{2\pi }{365}J)$$ (3)

$$\delta =0.409sin(\frac{2\pi }{365}J-1.39)$$ (4)

$$\omega_{s}=\arccos\left[-\tan\left(\varphi \right)\tan\left(\delta \right)\right]$$ (5)

The recommended value for the parameter is A = 0.16 for inland regions and A = 0.17 for coastal regions [1]. All statistical models used in this study yield estimates of total daily solar irradiation, in MJ/m^2^ day. These values were multiplied by 11.6 to convert to average irradiance, in W/m^2^, which is the quantity reported by the weather stations.

#### 2.3.2 Bristow & Campbell (B&C) model

This model uses the following two equations:

$$R_{s}=R_{a}A[1-\exp\left(-B\Delta T^{C}\right)]$$ (6)

$$\Delta T({}^{\circ}C)=T_{\max i}-(T_{\min i}+T_{\min\left(i+1\right)})/2$$ (7)

The change in temperature is calculated as the difference between a day’s maximum temperature and the minimum temperature of the same day (generally early morning, local time) and the following day. The general interpretation is that the A parameter represents the average clear-sky transmissivity, and parameters B and C are dependent on the recorded air temperature [1]. The accuracy and simplicity of this model has made it great source of solar radiation estimates at sites that have no data available [1].

### 2.3.3 Donatelli & Campbell model

The Donatelli & Campbell model [23] is a form of the B&C model, and uses the following equations:

$$R_{s}=R_{a}A\left[1-\exp\left(-Bf(T_{avg})\Delta T^{2}\exp\left(\frac{T_{min}}{C}\right)\right)\right]$$ (8)

$$f(T_{avg})=0.017exp(\exp\left(-0.053T_{avg(i)}\right))$$ (9)

$$T_{avg(i)}({}^{\circ}C)=(T_{\max\left(i\right)}+T_{\min\left(i\right)})/2$$ (10)

The change in temperature (ΔT) is calculated using Eq (7).

#### 2.3.4 Goodin model

The Goodin model is another version of the B&C model, and was calibrated and tested using thirty years of data from Kansas, USA, and developed for use in crop models [7].

$$R_{s}=A*R_{a}*\left(1-\exp\left(-B*(\frac{\Delta T^{C}}{R_{a}})\right)\right)$$ (11)

The change in temperature (ΔT) is also calculated using Eq (7).

The modified B&C (Goodin) [6] model produced more accurate estimates than the simple B&C model when applied in Kansas [7]. The Goodin model also “provided reasonably accurate estimates of irradiance at non-instrumented sites” when applying it to data from Spain [1]. This further supported the decision to use the Goodin model, as it could lead to potential use at non-instrumented sites in Ethiopia.

### 2.4 Analysis-based radiation estimates

Analysis datasets—GDAS and MERRA-2—were applied to generate incoming solar radiation estimates in three ways. First, the standard incoming surface solar radiation variable from each analysis product was extracted for each study site. Second, the analysis was downscaled from each product’s native resolution (0.25° for GDAS during the period of analysis, and 0.5° x°0.625 for MERRA-2) to 5 km horizontal resolution using a slope-aspect correction adopted from Dingman [29] to account for the influence of sun angle on incoming direct radiation, and these slope-aspect corrected radiation values were extracted for each site. Third, to mimic the station-based empirical method, a hybrid approach was used in which the GDAS and MERRA-2 2 m air temperature fields were used to estimate surface radiation with the Goodin empirical model. The purpose of these comparisons was to assess a range of ways in which analysis data can be used to generate estimates of *R*_*S*_.

### 2.5 Statistical evaluation

To compare the solar radiation estimates to the *in situ* data, R^2^, RMSE, and MBE were calculated with the following equations [1]:

$$R^{2}=1-\sum \frac{{\left(R_{s,e}–R_{s,m}\right)}^{2}}{{\left(R_{s,m}-R_{avg,m}\right)}^{2}}$$ (12)

$$RMSE={\left[\frac{1}{N_{obs}}\sum {\left(R_{s,e}-R_{s,m}\right)}^{2}\right]}^{0.5}$$ (13)

$$MBE=\frac{1}{N_{obs}}\sum \left(R_{s,e}-R_{s,m}\right)$$ (14)

N_obs_ is the number of data points, R_s,m_ is the measured shortwave radiation, R_s,e_ is the estimated shortwave radiation, and R_avg,m_ is the average measured radiation.

## 3.0 Results

### 3.1 Empirical model comparison

The average of the parameters across all four sites was used to generate the shortwave radiation estimates (Table 2).

**Table 2:** The parameters fit for each model and each station.

**Hargreaves & Samani**	**A**	**B**	**C**
Kurar	0.17		
Yejubie	0.15		
Enebi	0.16		
Debre Markos	0.17		
Average	0.16		
**Bristow & Campbell**	**A**	**B**	**C**
Kurar	0.70	0.02	1.86
Yejubie	0.63	0.08	1.25
Enebi	1.04	0.04	1.16
Debre Markos	1.90	0.08	0.59
Average	1.07	0.06	1.21
**Donatelli & Campbell**	**A**	**B**	**C**
Kurar	0.70	0.42	33.30
Yejubie	0.57	0.30	13.64
Enebi	0.69	0.33	27.95
Debre Markos	0.63	0.39	16.07
Average	0.65	0.36	22.74
**Goodin**	**A**	**B**	**C**
Kurar	0.71	1.01	1.74
Yejubie	0.61	2.60	1.33
Enebi	0.89	1.91	1.19
Debre Markos	0.79	5.99	0.84
Average	0.75	2.88	1.27

The maximum, minimum, mean, and standard deviation was determined for each model, as well as the correlation to *in situ* data, RMSE and MBE (Table 3).

**Table 3:** The maximum, minimum, mean, standard deviation, correlation coefficient, R^2^, RMSE and MBE for the solar radiation estimates generating using the Hargreaves & Samani, Bristow & Campbell, Donatelli & Campbell, and Goodin models.

Method	Station name	Maximum (W/m^2^)	Minimum (W/m^2^)	Mean (W/m^2^)	SD (W/m^2^)	r	RMSE (W/m^2^)	MBE (W/m^2^)
Hargreaves & Samani	Kurar	271.8	125.0	221.7	25.1	0.65	42.1	-17.5
	Yejubie	282.5	117.4	224.8	29.2	0.60	42.6	19.7
	Enebi	294.8	100.0	224.6	29.2	0.59	37.6	-9.2
	D. Markos	293.9	113.6	217.1	25.9	0.61	43.0	-1.5
	**Average**	**285.7**	**114.0**	**222.0**	**27.4**	**0.61**	**41.3**	**-2.1**
Bristow & Campbell	Kurar	366.0	108.3	294.8	44.4	0.72	66.0	55.5
	Yejubie	388.9	95.2	297.4	51.1	0.60	102.2	92.3
	Enebi	396.6	98.7	299.4	50.0	0.59	37.6	65.6
	D. Markos	392.9	120.0	288.1	44.3	0.65	81.0	69.5
	**Average**	**386.1**	**105.6**	**294.9**	**47.5**	**0.64**	**71.7**	**70.8**
Donatelli & Campbell	Kurar	275.2	53.4	226.7	36.0	0.73	36.1	-12.6
	Yejubie	275.2	41.1	222.3	40.1	0.61	42.8	17.2
	Enebi	277.9	44.1	222.3	40.7	0.57	41.6	-11.5
	D. Markos	277.2	59.9	216.1	36.5	0.63	41.7	-2.5
	**Average**	**276.4**	**49.6**	**221.8**	**38.4**	**0.64**	**40.5**	**-2.3**
Goodin	Kurar	292.8	100.5	249.3	30.5	0.74	35.6	10.1
	Yejubie	303.3	88.2	250	34.4	0.62	58.6	45.8
	Enebi	307.2	91.3	250.7	34.2	0.57	41.5	16.9
	D. Markos	305.7	111.1	245.6	29.9	0.68	47.1	27.7
	**Average**	**302.3**	**97.7**	**248.9**	**32.2**	**0.65**	**45.7**	**25.1**

The Bristow & Campbell model (Table 3) produced the largest RMSE, mean bias error, and standard deviation out of all the temperature-based models tested. The differences in correlation between the four tested models were small. However, the Goodin model statistical results fell more in the middle, as it produced maximum, minimum, standard deviation, RMSE and bias error values that were not extremely large or small compared to the other models. For this reason, and due to its demonstrated potential at non-instrumented sites [1], the Goodin model was selected to generate the empirical radiation estimates. All of the following empirical results discussed refer to estimates generated using the Goodin model.

### 3.2 Temperature comparison

**Fig 2:**
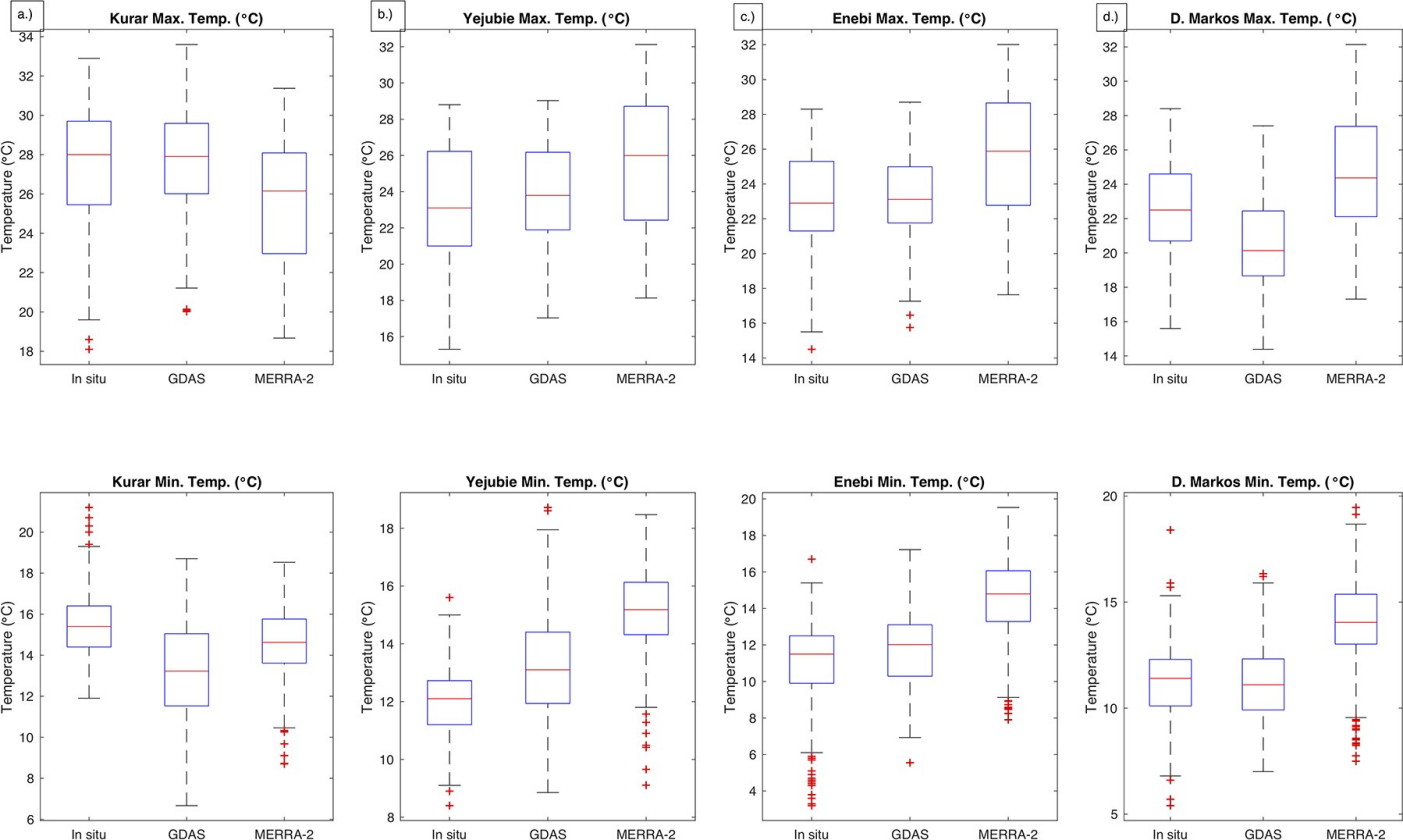
Box plots to show distribution and variability in *in situ* and reanalysis maximum and minimum temperature data for a.) Kurar, b.) Yejubie, c.) Enebi, and d.) Debre Markos.

The empirical solar radiation estimates were calculated using daily maximum and minimum air temperature. Therefore, we examined the temperature station data and compared it to GDAS and MERRA-2 records. The GDAS and MERRA-2 temperature values did not change when applying the slope-aspect correction. The maximum, minimum, mean, and standard deviation are shown in Fig 2, while the correlation, RMSE, and MBE of each set of temperature estimates for each station are reported in Table 4.

**Table 4:** The correlation coefficient, RMSE, MBE, and standard deviation for each station and the average across all four sites for maximum and minimum temperature.

GDAS–Maximum Temperature					
	Kurar	Yejubie	Enebi	D. Markos	**Average**
r	0.78	0.80	0.82	0.88	**0.82**
RMSE (°C)	1.90	1.9	1.6	1.6	**1.74**
MBE (°C)	0.13	0.5	0.2	-2.1	**-0.31**
Standard deviation (°C)	2.5	2.6	2.2	2.6	**2.5**
MERRA-2 –Maximum Temperature					
	Kurar	Yejubie	Enebi	D. Markos	**Average**
r	0.81	0.82	0.87	0.86	**0.84**
RMSE (°C)	2.66	3.0	3.2	2.9	**2.94**
MBE (°C)	-1.91	2.2	2.7	2.3	**1.31**
Standard deviation (°C)	3.1	3.7	3.5	3.5	**3.4**
GDAS–Minimum Temperature					
	Kurar	Yejubie	Enebi	D. Markos	**Average**
r	0.68	0.51	0.69	0.72	**0.65**
RMSE (°C)	2.90	2.0	2.0	1.3	**2.04**
MBE (°C)	-2.31	1.2	0.9	-0.1	**-0.10**
Standard deviation (°C)	2.4	1.9	2.0	1.8	**2.0**
MERRA-2 –Minimum Temperature					
	Kurar	Yejubie	Enebi	D. Markos	**Average**
r	0.60	0.54	0.81	0.74	**0.67**
RMSE (°C)	1.82	3.5	3.8	3.0	**3.03**
MBE (°C)	-0.98	3.2	3.5	2.7	**2.11**
Standard deviation (°C)	1.8	1.5	2.2	2.1	**1.9**

Overall MERRA-2 tends to produce warmer maximum and minimum temperature values than GDAS and the station recorded data (Fig 2). This is also reflected in the mean bias, as GDAS has a smaller, negative average bias while MERRA-2 has a larger, positive (warm) bias (Table 4). MERRA-2 has larger RMSE than GDAS for both maximum and minimum temperatures. MERRA-2 is more variable than GDAS for the maximum temperature, but the difference in standard deviation is not as large for the minimum temperature. In terms of correlation, MERRA-2 and GDAS produce similar values. The only large difference in correlation values is for the minimum temperature at Enebi (Table 4).

### 3.3 Solar radiation estimates

**Fig 3:**
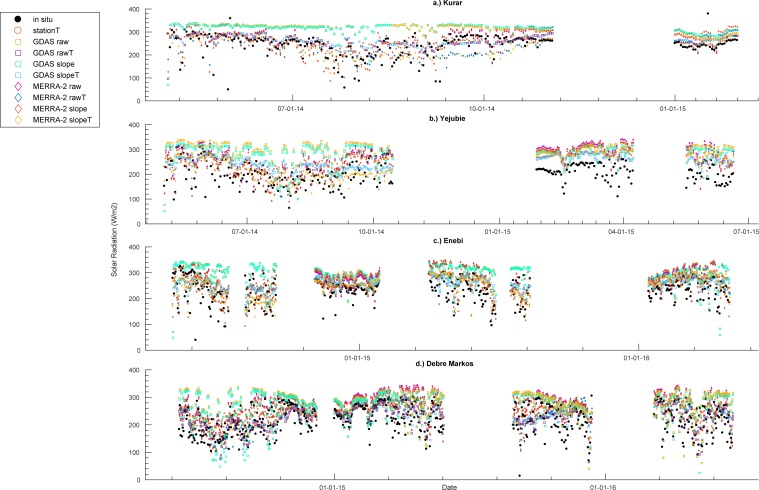
Solar radiation estimates and in situ data for a.) Kurar, b.) Yejubie, c.) Enebi, and d.) Debre Markos stations.

Due to limitations in field access to download data, particularly outside of the major cropping seasons, the time records at the four weather stations are discontinuous. Nevertheless, some seasonal and sub-seasonal patterns are evident in the solar radiation record (Fig 3). In most but not all cases there is a dip in solar radiation during the wet season (June-September), though the cloudiness effect is offset by the fact that the stations are in the Northern Hemisphere and so get a slight increase in potential incoming solar radiation in boreal summer. To first order, this temporal variability is captured by empirical methods and appears to be captured by MERRA-2. GDAS tends to underestimate seasonal variability, though hybrid estimates that use GDAS temperature and empirical solar radiation algorithms do capture seasonal effects.

**Fig 4:**
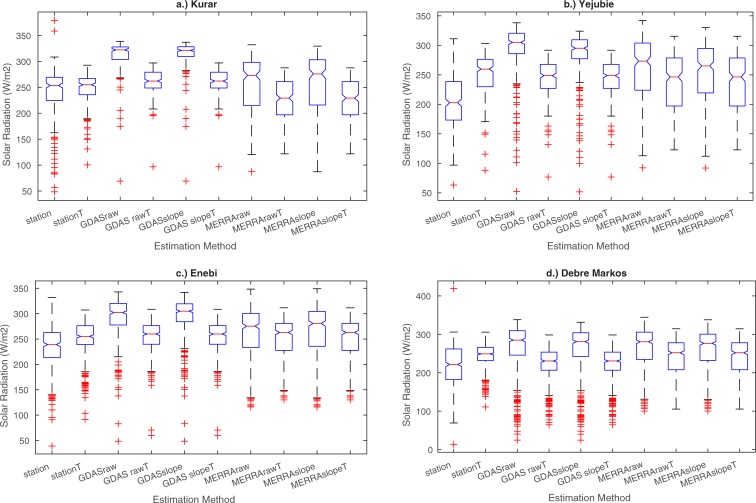
Box plots to show distribution and variability in *in situ* data and each set of solar radiation estimates for a.) Kurar, b.) Yejubie, c.) Enebi, and d.) Debre Markos.

**Fig 5:**
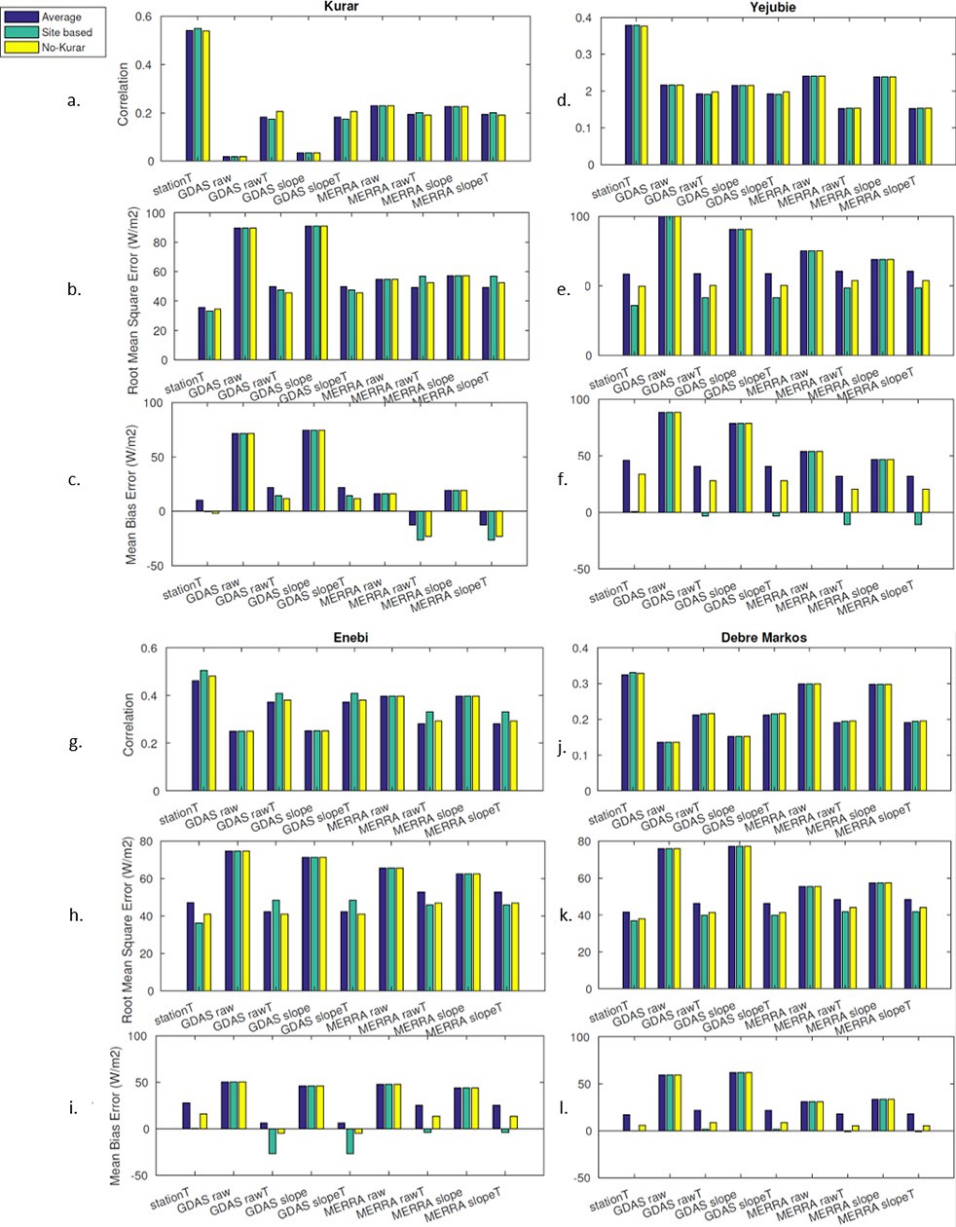
The correlation, RMSE and MBE for each set of parameters for Kurar (a-c), Yejubie (d-f), Enebi (g-i), and Debre Markos (j-l).

We also see that GDAS has the highest *R*_*S*_ estimates (Fig 4), and that these estimates are biased high relative to station observations (Fig 5). MERRA-2 *R*_*S*_ also tends to exceed observed values, though not as severely as GDAS (Fig 4). Empirical estimates based on *in situ* temperature measurements perform much better in terms of bias but underestimate the range of observed values. In comparison, MERRA-2 does not underestimate the range of values, but has larger bias. The hybrid estimates that utilize the analysis system temperatures (both GDAS and MERRA-2) have a reduction in range values compared to the direct radiation outputs.

### 3.4 Evaluation of radiation estimates

For all stations, the empirical model based on *in situ* station temperature measurements (*stationT*) provided the highest correlation with observed *R*_*S*_ (Table 5, blue bars in Fig 5). For Kurar, which is located on steep topography that poses a challenge for relatively coarse analysis products, this advantage was dramatic. *StationT* was better than almost all other methods by a statistically significant margin (p < 0.05, after accounting for autocorrelation) with only MERRA-2 radiation estimates (both with and without slope correction) coming slightly closer to *stationT* performance. At the other sites the analysis-based estimates fared better relative to *stationT*; while *stationT* always provided the highest correlation, MERRA-2 with and without slope correction performed almost exactly as well as *stationT* at the flat, plain site of Enebi, and it performed nearly as well as *stationT* in the rolling topography of the Debre Markos and Yejubie sites. Interestingly, *R*_*S*_ estimates taken directly from MERRA-2 (either with or without slope correction) tended to provide higher correlation with observation than estimates derived from MERRA-2 temperature, but the opposite was true for GDAS. There is no obvious reason why MERRA-2 *R*_*S*_ would outperform GDAS at these sites, but it is intuitive that GDAS temperature-based estimates would fare relatively well on account of the lower temperature bias in GDAS at most of the sites (Table 4).

**Table 5:** Correlation (R^2^) with in situ data for each station and the average correlation across all four sites. The top performing method that does not use *in situ* data are shown in bold. Italicized entries are significantly worse (p<0.05) than *stationT*. The correlation coefficients were transferred to z scores using Fisher's r to z transformation, and the effective sample size was used.

	Kurar	Yejubie	Enebi	D. Markos	Average
stationT	0.54	0.38	0.32	0.46	0.43
GDAS raw	*0*.*02*	0.22	*0*.*14*	*0*.*25*	0.16
GDAS rawT	*0*.*18*	0.19	0.21	0.37	0.24
GDAS slope	*0*.*04*	0.22	*0*.*15*	*0*.*25*	0.16
GDAS slopeT	*0*.*18*	0.19	0.21	0.37	0.24
MERRA raw	***0*.*23***	**0.24**	**0.30**	**0.40**	**0.29**
MERRA rawT	*0*.*19*	*0*.*15*	0.19	*0*.*28*	0.20
MERRA slope	***0*.*23***	**0.24**	**0.30**	**0.40**	**0.29**
MERRA slopeT	*0*.*19*	*0*.*15*	0.19	*0*.*28*	0.20

The solar radiation estimates taken from GDAS had the largest mean bias error across all stations (Fig 5). There was also a decrease in MBE when using the temperature-based estimates instead of the system radiation outputs (for both GDAS and MERRA-2). The decrease in bias error was more noticeable for GDAS, which complements the observed improvement in correlation (Table 5) and RMSE (Fig 5) when using GDAS temperature-based estimates instead of the direct GDAS radiation values. The MERRA-2 temperature-based estimates produced a negative bias in Kurar, possibly due to the warm temperature bias.

Similar to the mean bias results, GDAS had the largest root mean square error values across all stations (Fig 5). For both GDAS and MERRA-2, there was a decrease in RMSE when using the temperature-based estimates instead of the direct radiation outputs. Furthermore, the GDAS and MERRA-2 temperature-based estimates (*GDAS/MERRA-2 rawT* & *slopeT*) produced similar RMSE values, and *GDAS rawT/slopeT* had RMSE values slightly closer to the empirical model *in situ* temperature-based estimates (*stationT*). It is understandable that the two systems’ temperature-based estimates produced similar RMSE values since they performed comparably for the air temperature predictions (Fig 2 & Table 4). The proximity in the RMSE values for GDAS and stationT could be explained by the lower temperature bias for GDAS than MERRA-2 (Table 4). Similar to the correlation, the improved performance of *stationT* estimates was most noticeable at Kurar, with it having the smallest RMSE.

#### 3.4.1 Influence of parameters on estimates

The results reported above were generated using the average of the coefficients at all sites for the temperature-based estimates of solar radiation (see Table 2). To test sensitivity to local coefficients, we repeated the calculations using the site-specific coefficients.

The ‘site-based’ results (green bars in Fig 5) showed that there are very small decreases in correlation when using the average of the site-based coefficients for all locations. However, there are more noticeable changes in mean bias when using the site-based coefficients. All of the empirical model estimates have an improvement in MBE at the Yejubie and Enebi stations when using the individual site parameters. At the Kurar station, there is a reduction in mean bias error for the *stationT* and GDAS temperature-based estimates, but an increase in MBE for MERRA-2 temperature-based estimate (Fig 5C). For the Enebi station, *stationT* and MERRA-2 temperature-based estimates have an improvement in mean bias error, while the GDAS temperature-based estimates have an increase of mean bias error.

The Kurar station parameters were noticeably different from the other station parameters (the smallest B parameter at 1.01, and the largest C parameter at 1.74, see Table 2). Considering the difference in coefficients and the fact that the Kurar station is located at the lowest elevation of the test sites, we recalculated the average coefficients using the parameters from the Yejubie, Enebi and Debre Markos stations and applied them to the empirical models for all of the stations (referred to as ‘no-Kurar’). The new average of the parameters was: A = 0.76, B = 3.50, C = 1.12. The R^2^, RMSE and MBE were also calculated. The ‘no-Kurar’ results are represented as the yellow bars in Fig 5, and were compared to the blue bars (original average of parameters) in the same figure. There are small increases in correlation for the higher elevation sites (Debre Markos and Enebi). Similar to the site-based coefficient analysis, there are improvements in mean bias error. The Yejubie and Debre Markos stations have improvements in MBE when compared to the original average of parameters (but not as large of an improvement as the site-based coefficients) for all sets of empirical model estimates (Fig 5F and 5L). The *stationT* and GDAS temperature-based values have an improvement at Kurar, but MERRA-2 has an increase in MBE (Fig 5C). At the Enebi station, the station temperature and MERRA-2 model estimates have improved MBE, as well as a small improvement in MBE for GDAS temperature estimates (6.38 W/m^2^ to -4.83 W/m^2^). These coefficient sensitivity tests suggest that estimates of temporal variability in *R*_*S*_, as reflected in the correlation with observations at each site, are relatively insensitive to empirical coefficients. Mean bias, however, is meaningfully affected by choice of coefficients.

## 4.0 Discussion

### 4.1 Temperature comparison

The GDAS maximum and minimum temperature predictions typically had a smaller standard deviation than MERRA-2 temperature values. Since MERRA-2 had a larger variation in temperature estimates, one thought that could be considered is that MERRA-2 could be more capable of capturing Ethiopia’s complex climate and temperature range. Despite the large standard deviation, however, MERRA-2 tended to have warmer temperatures than the station data. Furthermore, GDAS had smaller bias and root mean square error than MERRA-2. Having a larger standard deviation or a reduced bias and error does not seem to have a strong relationship with the model’s ability to capture temperature variability, as GDAS and MERRA-2 performed comparably in this region when comparing correlation values. Despite the complexity of the region’s climate and topography, both analysis systems capture the temporal pattern of variability about equally as well.

There is a general agreement that these temperature-based empirical models can make reliable solar radiation estimates [1, 20]. However, one factor that should be taken into consideration is that the temperature-based empirical models assume that air temperature is the dominant factor that influences incoming solar radiation [7]. Other factors could muddle the relationship between air temperature and incoming shortwave radiation, such as topography, wind speed, water vapor, and weather systems, and therefore influence the accuracy of temperature-based empirical models at higher elevations [4]. These complicating factors may be particularly relevant in a tropical highland region, where convection can be intense and highly localized.

### 4.2 General patterns in solar radiation estimation comparison

The GDAS estimates had the highest means across all four stations, but smaller standard deviations, larger RMSE and mean bias, as well as lower correlations to the in situ data. The GDAS solar radiation estimates were generally too large, and limited to a higher range of values. The improvements in correlation and reduction in RMSE and MBE when using the temperature-based estimates from GDAS instead of the direct radiation values indicated that if planning to obtain solar radiation estimates from GDAS, it is better to use the predicted air temperature in the empirical model than to use the shortwave radiation estimates. MERRA-2 also had large mean values (when compared to the mean of the *in situ* data), but generally had large standard deviations, which seemed to make the estimates more comparable to station radiation values for this region.

When comparing the two best methods (in terms of correlation), MERRA-2 had a range similar to the station data, but also had a large bias. The *stationT* estimates had a limited range in radiation values, but a smaller bias. Using the temperature estimates from MERRA-2 (or GDAS) in the empirical model could be seen as a middle ground, as it reduced the range of values, but it also improved the mean bias. When just looking at correlation values, MERRA-2 performed comparably to *stationT* at the Enebi station, and also performed well at the Debre Markos and Yejubie stations. There was only a drastic difference in correlation between those two sets of estimates at the Kurar station, which was located in the steepest terrain of the four sites. This could indicate that MERRA-2 performs better in areas of flatter topography. In contrast, the correlation of *stationT* decreased at the higher elevation sites. This could suggest that the empirical model does not perform well in areas of higher elevation, possibly due to its limited range of radiation values. The complex climate and elevation profile in the Ethiopian Highlands most likely challenged the ability of reanalysis systems to make accurate solar radiation predictions in this region.

### 4.3 Raw output v. slope corrected output of GDAS and MERRA-2

The differences between the mean and standard deviation of the raw and slope-corrected outputs of GDAS and MERRA-2 were small. The changes in correlation between the raw output and slope-corrected outputs of the analysis systems were also minimal. There was essentially no change in correlation in the MERRA-2 sets of estimates, while there were very small changes in correlation at the Kurar and Enebi stations for the GDAS estimates. While there were no large changes in correlation when comparing the raw output and the slope-corrected output of the reanalysis systems, there were slight changes in mean bias error (Fig 5). At the Kurar and Enebi stations, the raw output of both GDAS and MERRA-2 had slightly improved MBE, while at the Yejubie and Debre Markos stations, the slope-corrected output of GDAS and MERRA-2 had improved MBE. A possible explanation for this is related to the topography at the site locations. It is reasonable that the raw output of these systems would have improved mean bias error at the Enebi station since there is no slope in the topography there. The Kurar station is located in the steepest terrain, so it is possible that the slope-corrected output does not capture highly localized topography, resulting in the raw output having better estimates. The Yejubie and Debre Markos stations are both located in the rolling hills, which have a moderate change in topography and therefore the slope correction is able to provide more accurate solar radiation estimates.

### 4.4 Site-based coefficients and leave-out analysis

It is reasonable that there was a slight improvement in correlation at the higher elevation sites (Debre Markos and Enebi) when the Kurar parameters (the lowest elevation site) were not considered in the average. The very small improvement indicated that using the average coefficients did not cause a large decrease in accuracy of shortwave radiation estimates. While the changes in correlation when using the site-based coefficients were small, there were some notable changes in mean bias error. These changes in mean bias error also occurred when conducting the ‘no-Kurar’ analysis. The increase in MBE for the MERRA-2 temperature estimates at the Kurar station (for both the site-based and the ‘no Kurar’ tests) supports the idea that MERRA-2 does not perform well in locations of steep topography. Though there was an improvement in mean bias error at the Debre Markos station, which is located in the hills region of Ethiopia, possibly suggesting that MERRA-2 can handle some smaller changes in topography.

## 5.0 Conclusions

Solar radiation estimates are a critical component of many scientific analyses, but *in situ* data can be hard to obtain. Empirical temperature-based models provide one way of generating accurate estimates with easily obtainable data. Meteorological analysis systems such as GDAS and MERRA-2, also serve as a source of solar radiation values. The goal of this study was to see which source of estimates provided the most reliable shortwave radiation values for a tropical highland region.

*StationT* produced the highest correlations to *in situ* data, but the MERRA-2 raw outputs also performed well. It appeared that *stationT* performed best in the areas of lower elevation, while the raw output of MERRA-2 had improved solar radiation estimates in areas of higher elevation. It is possible that topography played a role, as MERRA-2 performed comparably to *stationT* at the Enebi (flat plain), Debre Markos and Yejubie (rolling hills) stations, but not so at the Kurar station (steepest terrain).

This study provides a basis for selecting a shortwave radiation estimate when applying crop models, studying land-atmosphere interactions, or mapping solar power potential in the Ethiopian highlands. The comparisons performed here can also inform work elsewhere in the highland tropics, where consistent in situ solar radiation observations are rarely available. Future work could include expanding the testing range beyond the highlands to other regions of Ethiopia. Since the climate of Ethiopia is complex, future research could also include testing other empirical models that use other weather variables such as rainfall, though this is dependent on the records available at the weather stations.
